# Unicentric mixed variant Castleman disease associated with intrabronchial plasmacytoma

**DOI:** 10.1186/1746-1596-9-64

**Published:** 2014-03-20

**Authors:** Noémi Eszes, Lilla Tamási, Attila Csekeő, Judit Csomor, Ágota Szepesi, Gergely Varga, György Balázs, György Losonczy, Veronika Müller

**Affiliations:** 1 Department of Pulmonology, Semmelweis University, Diósárok u. 1/c, 1125 Budapest, Hungary; 2 Koranyi National Institute for Tuberculosis and Pulmonology, Budapest, Hungary; 3 1st Department of Pathology and Experimental Cancer Research, Semmelweis University, Budapest, Hungary; 4 3rd Department of Internal Medicine, Semmelweis University, Budapest, Hungary; 5 Department of Diagnostic Radiology, Heart Center, Semmelweis University, Budapest, Hungary

**Keywords:** Castleman disease, Unicentric, Mixed variant, Extramedullary plasmacytoma, Intrabronchial plasmacytoma

## Abstract

**Virtual slides:**

The virtual slide(s) for this article can be found here: http://www.diagnosticpathology.diagnomx.eu/vs/2872096831190851.

Castleman disease (CD), described as a heterogeneous lymphoproliferative disorder, can be divided into different subtypes according to clinical appearance (unicentric and multicentric form) and histopathological features (hyaline vascular, plasma cell, mixed type, human herpesvirus 8–associated and multicentric not otherwise specified). Unicentric CD is known to be usually of the hyaline vascular variant, plasma cell and mixed type of this form are quite uncommon. Malignancies are mainly associated with the multicentric form. We report a rare case of unicentric mixed variant CD evolving into intrabronchial, extramedullary plasmacytoma.

Intrabronchial mass with consequential obstruction of the left main bronchus, left lung atelectasis and mediastinal lymphadenomegaly was detected by chest CT in our patient suffering from cough and hemoptysis. Pulmonectomy was performed, histopathological and immunhistochemical analysis of lymph nodes revealed mixed type of CD with interfollicular monotypic plasma cell proliferation. The intrabronchial mass consisted of monotypic plasma cells confirming plasmacytoma. Systemic involvement was not confirmed by further tests.

Although malignancies more often present in multicentric CD that usually belongs to the plasma cell subtype, this case confirms the neoplastic potential of the rarest, unicentric mixed variant of CD.

## Background

Castleman disease (CD) - also known as giant lymph node hyperplasia or angiofollicular lymph node hyperplasia - has a wide spectrum of appearances due to its heterogeneous pathological and clinical characteristics. It may appear in any part of the body as nodal or extranodal mass.

Regarding the clinical presentation, unicentric (localized) and multicentric (systemic) form can be identified. The unicentric type usually presents as a benign, asymptomatic disease affecting one single, or localized group of lymph nodes most often observed in the mediastinal region. Complete local surgical removal is curative in most cases.

Multicentric CD frequently associated with HIV infection and Kaposi’s sarcoma, is more aggressive and usually high risk for malignant transformation into lymphoma or other malignant lymphoproliferative diseases [1]. This form often occurs with systemic symptoms (such as fever, weight loss, night sweats, splenomegaly) and abnormal laboratory results (hypergammaglobulinemia, elevated liver enzymes, anemia, thrombocytopenia and elevated interleukin (IL)-6 levels) [2,3]. The prognosis is poor with an overall mortality of 50%.

During the past decades, the group of CD subtypes has been expanded. Histopathological classification has just been modified and updated [4]. According to the new classification, 5 forms can be distinguished: hyaline vascular, plasma cell, mixed type, human herpesvirus 8–associated and multicentric not otherwise specified CD.

The hyaline vascular type was first reported by Benjamin Castleman in 1956 [5]. In these cases the follicle centres often show atrophic changes with an increased mantle zone that has an “onion skin” pattern due to the small lymphocytes that are located in concentric circles. Penetrating vessels surrounded by hyalinized stroma and proliferation of the follicular dendritic cells may also be seen. The interfollicular region is highly vascularized and consists predominantly of small T lymphocytes [6].

In contrast, the plasma cell variant of the disease is characterized by follicular hyperplasia and polyclonal interfollicular sheets of proliferating mature plasma cells [7]. Occasionally immunoglobulin light chain (IgG or IgA lambda) restricted plasma cell populations were also observed [8,9].

There are casual cases described as “mixed type” in which the histopathologic features of both variants take place together suggesting a possible connection between the two forms.

Two new histological categories have been described recently: the human herpesvirus 8 (HHV-8)–associated (plasmablastic) multicentric CD, which presents most frequently in immunosuppressed (particularly in HIV-positive) patients, and another subgroup, which is defined as not otherwise specified multicentric CD with non-specific pathological findings, that have similar histologic features to that of plasma cell variant but that cannot be classified into either HHV-8 associated or into the plasma cell variant type [4].

Ninety percent of all cases belong to the hyaline vascular type of the disease, which is usually unicentric and has mostly an asymptomatic course but alternatively symptoms may occur due to localization [10]. On the contrary the less common plasma cell and mixed variants are in most of the cases multicentric and mainly manifest with systemic symptoms [6].

## Case presentation

A nonsmoking 51-year-old obese woman presented with cough and hemoptysis to our center for further examinations. Her medical history included panic disorder, hysterectomy due to myoma and gastroesophageal reflux disease with concomitant *Helicobacter* pylori positivity which was successfully eradicated. A few years ago she was diagnosed with asthma but did not take the medications prescribed.

**Figure 1:**
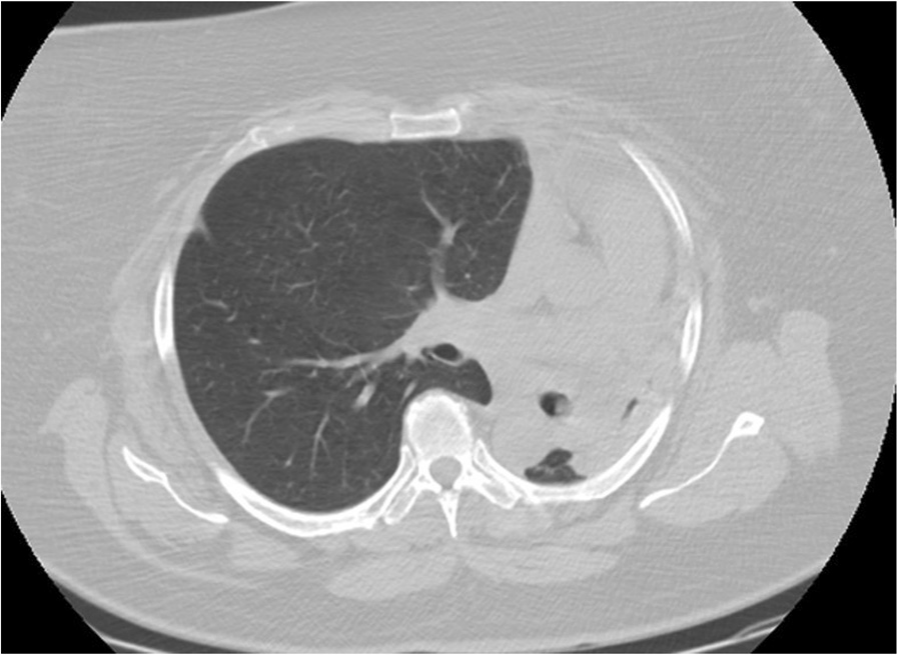
Chest CT image of total atelectasis of the left lung.

**Figure 2:**
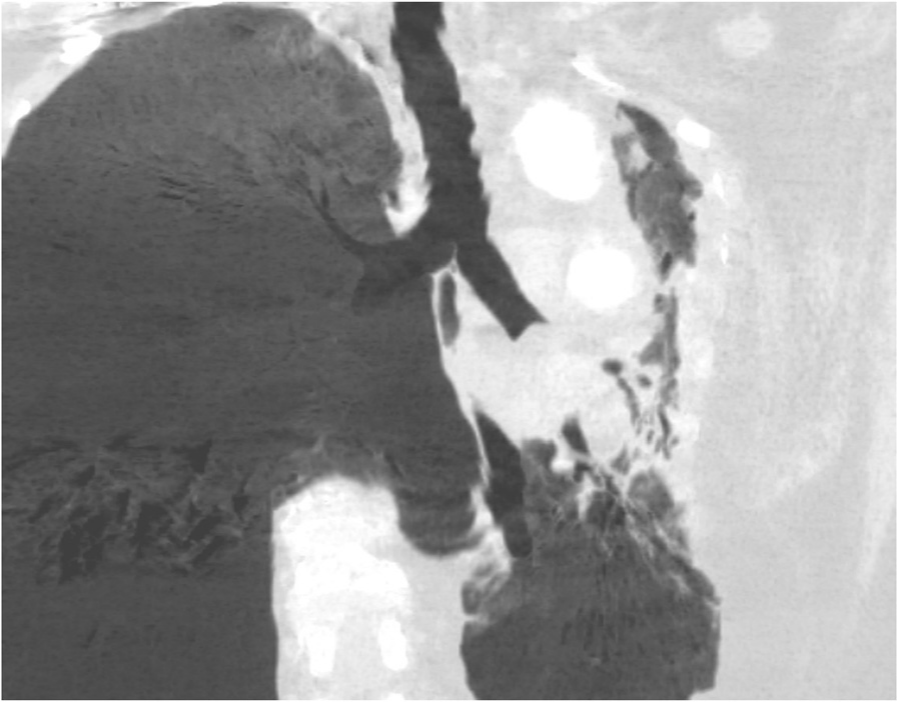
2 D chest CT image of the intrabronchial mass located 3 cm from the main carina.

**Figure 3:**
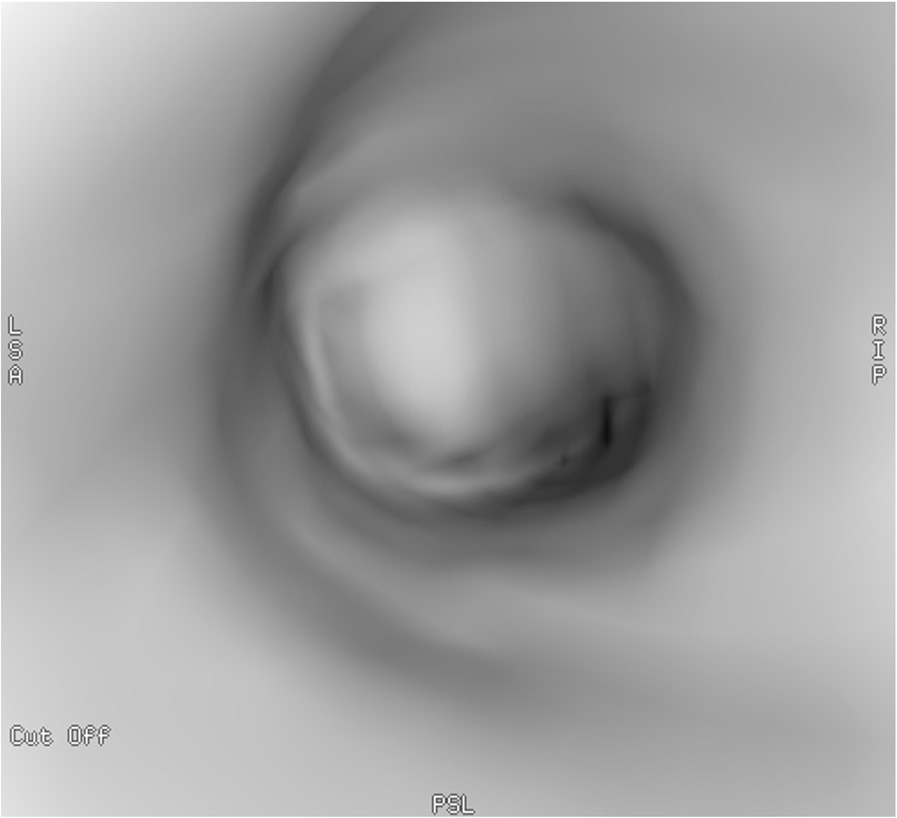
Image of virtual bronchoscopy: complete obstruction of the left main bronchus on the left side could be seen.

Chest X-ray showed total atelectasis of the left lung. In addition, chest CT revealed a soft-tissue density intrabronchial mass located only 3 cm from the main carina occluding the left main bronchus. Mediastinal deviation to the left could also be seen, but no hilar or mediastinal lymphadenopathy was observed (Figures 1 and 2). Right lung was intact without any parenchymal abnormality. Laboratory findings were within normal range except for slightly elevated level of Ca^2+^ (2.62 mmol/L; reference range: 2.10-2.42 mmol/L) erythrocyte sedimentation rate of 30 mm/h (reference range: 0–20 mm/h), a leukocytosis of 20000 cells/μL (reference range: 4000–10000 cells/μL) and slightly elevated level of uric acid of 474 μmol/L (reference range:0–340 μmol/L). Tumor markers also yielded normal results (CEA, AFP, CA 19–9, CA 72–4, CA 15–3, Cyfra 21–1, NSE, CA 125). Lung function test revealed dominantly restrictive ventilatory disorder (forced vital capacity (FVC): 2,5 L (85% predicted), forced expiratory volume in 1 second (FEV1):1,74 L (69% predicted), FEV1/FVC: 69%). Bronchoscopy could be performed only under general anesthesia because of glottic spasm. During the examination deformation of orifices with no endobronchial changes of the right side and - consistent with the CT findings- complete obstruction of the left main bronchus could be seen (Figure 3).

Severe, massive bleeding resulted following bronchial biopsy, so no further sampling of the lesion was possible. Histological examination of the obtained two samples showed groups of cells with small round nuclei in the inflamed bronchial mucosa and vimentin positivity. Definitive diagnosis could not be established. Abdominal ultrasonography and mammography also were negative. Perfusion scintigraphy showed only 8% perfusion of the left atelectatic lung. Repeated chest CT (2 months after the patient’s first presentation) revealed the same status as previously detected but this time with additional mediastinal lymphadenopathy.

**Figure 4:**
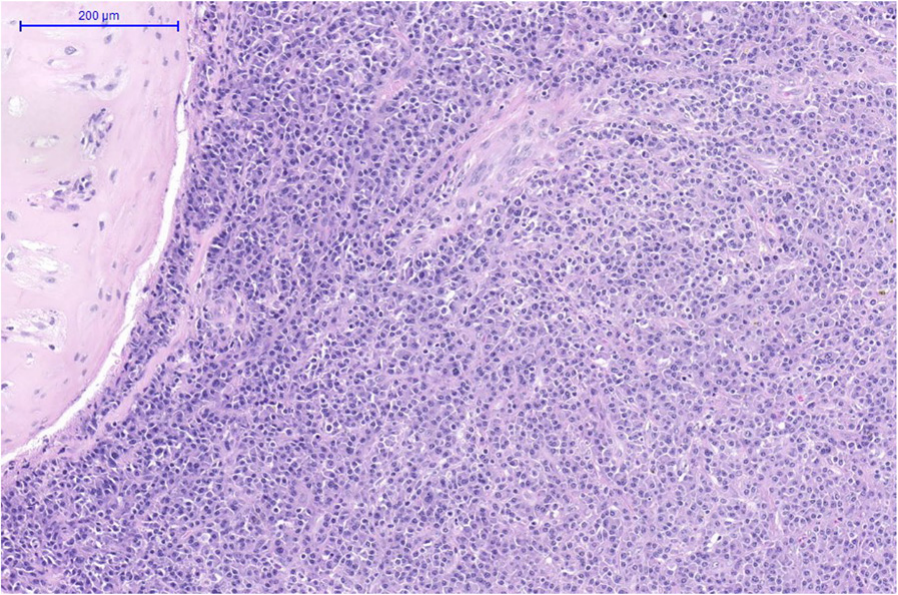
Intrabronchial plasmacytoma consisted of atypical plasma cells (HE magnification 100×).

**Figure 5:**
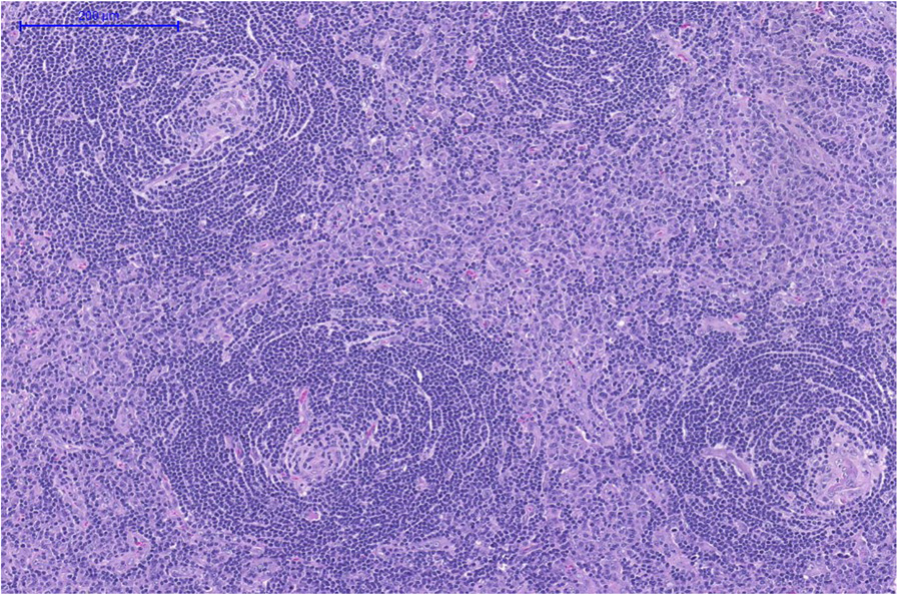
Castleman disease with small hyalinized germinal centers, concentric expansion of the mantle zones and interfollicular plasma cell infiltration (HE magnification100×).

**Figure 6:**
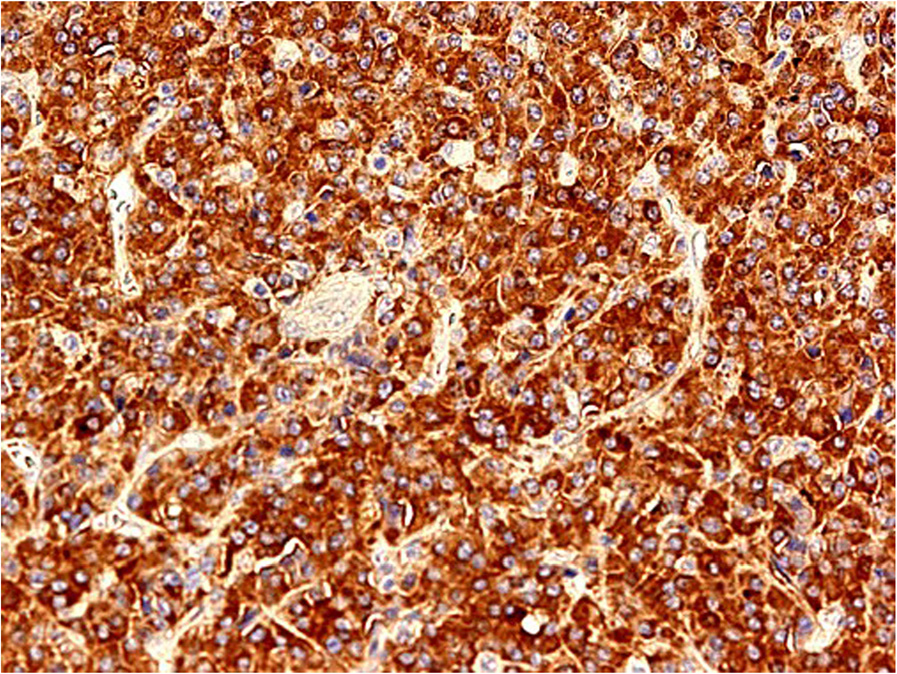
Monotypical lambda light chain restricted cells in plasmacytoma (magnification 400×).

**Figure 7:**
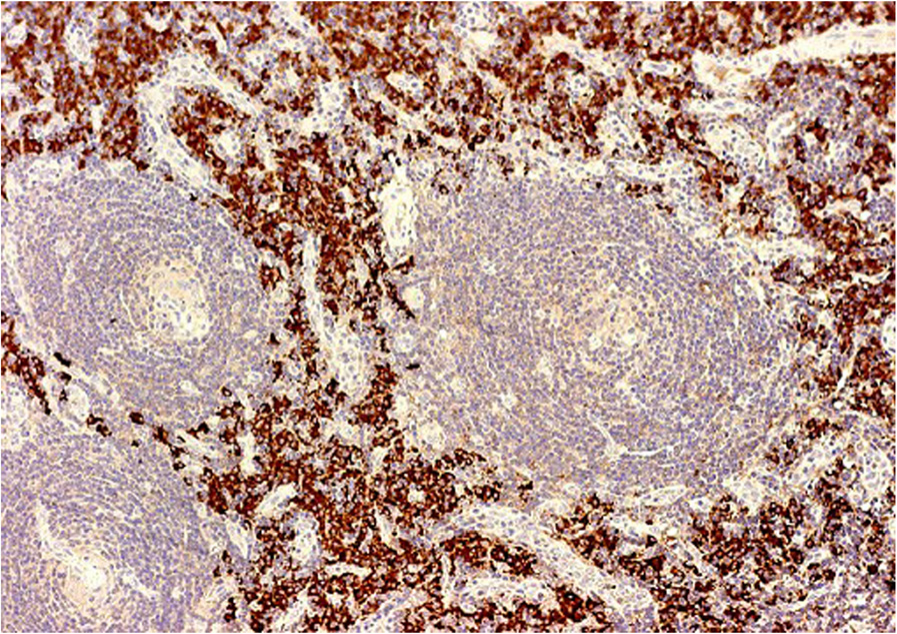
Mixed variant of Castleman disease with lambda monoclonal plasma cell infiltration (magnification 400×).

Patient underwent left side pulmonectomy with resection of the affected lymph nodes. Histological examination showed that the intrabronchial mass was comprised of monomorph mature plasma cells confirming endobronchial plasmacytoma. In peribronchial lymph nodes depleted germinal centres were revealed with expanded mantle zone showing the “onion skin” pattern due to the concentric layers of small lymphocytes. Interfollicular area consisted mainly of lymphocytes and interfollicular vascularisation. In other areas interfollicular sheets of mature plasma cells were shown with similar morphology as that of the plasma cells in the intrabronchial mass (Figures 4 and 5). Immunohistochemistry demonstrated the plasma cells to be positive for CD 31 with immunoglobulin lambda light chain restriction (Figures 6 and 7). These findings were consistent with the mixed variant of CD associated with endobronchial plasmocytoma. Systemic involvement was not confirmed by further hematological tests. Bone marrow aspiration showed no clonal plasma cell populations, biopsy could not be performed because of the patient’s obesity. Although serum protein electrophoresis with immunofixation showed the presence of monoclonal lambda light chain protein, it decreased rapidly after surgery. Urine test for Bence Jones protein was negative. A metastatic bone survey was performed including imaging X-rays of the skull, vertebral column, pelvis, and extremities. A lesion suspicious for metastasis of the right proximal femur was detected, therefore PET-CT scan was performed, but it did not confirm activity.

Tree years after surgery the patient is asymptomatic, with no signs of recurrence of the disease.

## Discussion

CD is usually reported as a rare benign disorder characterized by lymphocyte proliferation but several case reports published in the last decades proved its malignant potential. Malignancies are usually present with the multricentric form of CD including Kaposi sarcoma, B cell lymphoma, Hodgkin’s lymphoma and plasmacytoma. Other conditions were also observed accompanying mainly the multricentric form, e.g. POEMS syndrome (polyneuropathy, organomegaly, endocrinopathy, monoclonal gammopathy, and skin changes), nephrotic syndrome, amyloidosis, connective tissue diseases and other autoimmune disorders [11,12]. In the hyaline vascular type of CD, dendritic cell tumors and vascular neoplasms have been described most frequently. Besides, HHV8 positive CD, a special subtype of multicentric CD, may be associated with a special entity named “large B-cell lymphoma arising in HHV-8-associated multicentric CD” or “HHV-8-positive plasmablastic lymphoma” consisting of IgM lambda expressing immunoblasts that are located in the mantle zone. Interestingly, somatic hypermutation of immunoglobulin genes did not occur in this type of lymphoma [13].

Unicentric cases of CD followed by lymphoma or solitary plasmacytoma are quite spare and were reported only in a few cases [14]. Extramedullary plasmacytoma presenting simultaneously with the unicentric variant is even more uncommon, an intracranial form with hyaline-vascular type of CD was described previously [15]. Extramedullary plasmacytoma is an extremely rare entity of its own, takes up approximately 4% of all plasma cell tumors [16]. The lymphoid cells in CD of all pathologic types are mainly polyclonal. In some cases, lymph node extramedullary plasmacytoma arising in Castleman disease was diagnosed based on the presence of monotypic IgG or IgA λ plasma cell infiltration [17-20]. Of note, in patients suffering from CD with monotypic plasma cell infiltrates, cytogenetic analysis -detected by immonhistochemistry- does not always show any immunoglobulin gene rearrangements in the background [21,22]. The difference between plasma cell type CD with monoclonal plasma cells and lymph node plasmacytoma is not clear, it is still a question whether plasma cell variant CD with monoclonal plasma cells can be diagnosed as plasmacytoma. Diagnosis of plasma cell type CD with monoclonal plasma cells was recommended when some CD specific follicles can also be seen. Lack of these pathological characteristics substantiates the diagnosis of plasmacytoma [23].

The etiology of CD is not fully elucidated. The role of IL-6 cytokine may be one of the key components in the pathophysiology of the disease, its overexpression in germinal center cells of the lymph nodes was shown in the plasma cell type of CD [24]. In mice IL-6 induced histological and clinical changes were specific to CD [25]. IL-6 is responsible among others for acute phase response, CD4 T cell differentiation, B cell growth and differentiation into plasma cells. Its role was suggested in the development of B cell lymphomas and plasmacytomagenesis, thus it may be a link between plasmacytoma and plasma cell type of CD [26]. IL-6 also increases the production of hepcidin, which was described to be associated with iron deficiency anaemia in mixed type of CD [27]. Furthermore, IL-6 stimulates the expression of vascular epithelial growth factor (VEGF), causing vascular proliferation, which is thought to be of great importance in the development of vascular neoplasms in CD. In HHV8 associated CD, the virus encodes a viral homologue of IL-6, that induces the expression of VEGF [28] which may stimulate the human IL-6 production. This mechanism may participate in the development of the HHV8 associated multicentric CD. Recently, epidermal growth factor receptor (EGFR) overexpression was observed in both CD and follicular dendritic cell (FDC) sarcoma, thus EGFR was suggested to be a connection between hyaline vascular CD and FDC sarcoma [29].

The definitive diagnosis of CD is based on histology. The results should be carefully evaluated as other lymphoproliferative disorders, especially lymphomas with plasmablastic features may resemble to CD regarding the histopathologic features. Plasmablastic lymphomas are also often associated with HIV or HHV8 infections similar to multicentric CD, and even EBV (Epstein-Barr virus) may occur in both entity [30,31]. Low-grade mucosa-associated lymphoid tissue (MALT) lymphomas also need to be distinguished from plasma cell type of CD. B cells are known to be characterized by CD5 expression in the expanded mantle zones of CD. In contrast, CD5 positivity is unusual in MALT lymphomas except for some rare cases [32], that can even closely mimic CD [33]. For establishing the accurate diagnosis, careful histopathologic examinations are needed, immunohistological examinations and immunologic gene rearrangement analysis can be helpful in the differentiation.

The optimal treatment for patients with unicentric disease is surgical excision, which is usually curative irrespectively of the histological variant. If the disease is non-resecable, additional treatments - adjuvant steroid therapy and radiotherapy - were reported to be successful [34]. Therapeutic options for patients with multricentric disease are more variable including corticosteroids (given temporarily according to the degree of the symptoms), combination chemotherapy regimens, intravenous immunoglobulin, antiviral medications, anti–IL-6 therapy and even bone marrow transplantation.

In cases of EMP, radiotherapy was accepted in general to be the first-line treatment based on the radiosensitivity of the disease. Small lesions may be cured with surgery without radiotherapy. Radiotherapy should be only considered when residual local disease can be detected. In our case surgical excision was part of the diagnosis. In respect of our patient, there is no evidence of benefit of adjuvant radiotherapy. Surgery was curative, the patient has no recurrence of the disease after 3 years of follow-up.

## Conclusion

Unicentric CD with concomitant neoplasm is a rare condition. Plasma cell or mixed type CD with monoclonal proliferation is even more exceptional. We reported a unique association of unicentric, mixed type CD with monoclonal plasma cell infiltration associated with extramedullary, intrabronchial plasmacytoma proving the neoplastic potential of this entity. According to previous findings plasma cell malignancy may have developed due to the elevated IL-6 levels, although we have not measured it in our patient. The significance of monoclonality present in plasma cell or mixed type of CD is still not clear; however according to our case, surgical removal of the lesions might be curative. Further studies are warranted to analyze the clinicopathological and cytogenetic characteristics of this condition and follow up more cases.

## Consent

Written informed consent was obtained from the patient for publication of this Case Report and any accompanying images. A copy of the written consent is available for review by the Editor-in-Chief of this journal.

## Abbreviations

CD: Castleman disease; HIV: Human immunodeficiency virus; IL: Interleukin; HHV: Human herpesvirus; CEA: Carcinoembryonic antigen; AFP: Alfa fetoprotein; CA: Cancer antigen; NSE: Neuron specific enolase; FEV1: Forced expiratory volume; FVC: Forced vital capacity; IL: Interleukin; VEGF: Vascular epithelial growth factor; EGFR: Epidermal growth factor receptor; FDC: Follicular dendritic cell.

## Competing interests

The authors declare that they have no competing interests.

## Authors’ contributions

NE wrote the paper, VM and GL drafted the manuscript and revised it critically for important intellectual content, LT and GV, GB have been involved in drafting the manuscript, GV performed the hematological exams of the patient, AC performed the pulmonectomy, GB performed the chest CT exams and virtual bronchoscopy, JC and AS performed the histological examinations and immunophenotyping. All authors read and approved the final manuscript.
